# 1654. Analysis of Prescribing Patterns for Respiratory Tract Illnesses Following the Conclusion of an Education and Feedback Intervention

**DOI:** 10.1093/ofid/ofac492.120

**Published:** 2022-12-15

**Authors:** James J Harrigan, Keith W Hamilton, Leigh Cressman, Warren B Bilker, Kathleen Degnan, David Tran, Michael Z David, David A Pegues, Lauren Dutcher

**Affiliations:** University of the Hospital of Pennsylvania, Philadelphia, Pennsylvania; University of Pennsylvania Perelman School of Medicine, Philadelphia, Pennsylvania; University of Pennsylvania, Philadelphia, Pennsylvania; University of Pennsylvania Perelman School of Medicine, Philadelphia, Pennsylvania; University of Pennsylvania Perelman School of Medicine, Philadelphia, Pennsylvania; Independent Contractor, Philadelphia, Pennsylvania; University of Pennsylvania Perelman School of Medicine, Philadelphia, Pennsylvania; Hospital of the University of Pennsylvania, Philadelphia, Pennsylvania for the Centers for Disease Control and Prevention (CDC) Epicenters Program; University of Pennsylvania Perelman School of Medicine, Philadelphia, Pennsylvania

## Abstract

**Background:**

We previously conducted a study in primary care practices assessing the impact of an educational session paired with peer comparison feedback on antibiotic prescribing, demonstrating a reduction in overall prescribing for respiratory tract diseases (RTDs). However, the lasting effects of this intervention on antibiotic prescribing patterns without ongoing feedback are unknown.

**Methods:**

To study the long-term effects of this feedback on antibiotic prescribing, we analyzed prescribing trends for 14 months after the initial study. We collected encounter-level data, including patient and provider information, ICD-10 codes, and antibiotics prescribed. RTDs were grouped into tiers based on prescribing appropriateness: tier 1 (almost always indicated), tier 2 (may be indicated), and tier 3 (rarely indicated). A χ^2^ test was used to compare proportions of antibiotic prescribing between three time periods: pre-intervention, intervention, and post-intervention (following cessation of provider feedback). A mixed-effects multivariable logistic regression analysis was performed to assess the association between the period and antibiotic prescribing.

**Results:**

We analyzed 260,900 encounters (127,324 pre-intervention, 58,431 during the intervention, and 75,145 post-intervention) from 28 practices, with patient, provider and practice characteristics in Table 1. Rates of antibiotic prescribing for RTD visits were higher in the post-intervention period than the intervention period (28.9% vs 23.0%, p< 0.001), but remained lower than the 35.2% pre-intervention rate (Figure 1, p< 0.001). In multivariable analyses, the odds of receiving a prescription was higher in the post-intervention compared to the intervention period for tier 2 (OR 1.19, 95% CI 1.10–1.30, p< 0.05) and tier 3 (OR 1.20, 95% CI 1.12–1.30) indications, but was still lower when compared to the pre-intervention period for each tier (OR 0.66, 95% CI 0.59–0.73 for tier 2; OR 0.68, 95% CI 0.61–0.75 for tier 3) (Table 2).

Table 1 includes patient, provider, and encounter level demographics.

Table 2 includes the results of the multivariable analysis.

Figure 1 is a graph of proportion of encounters with an antibiotic prescribed over time. The time period associated with the intervention is highlighted and graphs are separated by tier of appropriateness of antibiotic prescribing associated with the encounter.

**Conclusion:**

The effects of this targeted educational and feedback program last beyond the intervention period, but without ongoing provider feedback there is a trend toward increased prescribing. Future studies are needed to determine optimal strategies to maintain the efficacy of this intervention.

**Disclosures:**

**Kathleen Degnan, MD**, Gilead: Grant/Research Support **Michael Z. David, MD PhD**, Contrafect: Grant/Research Support|GSK: Advisor/Consultant|Johnson and Johnson: Advisor/Consultant.

**Figure:**
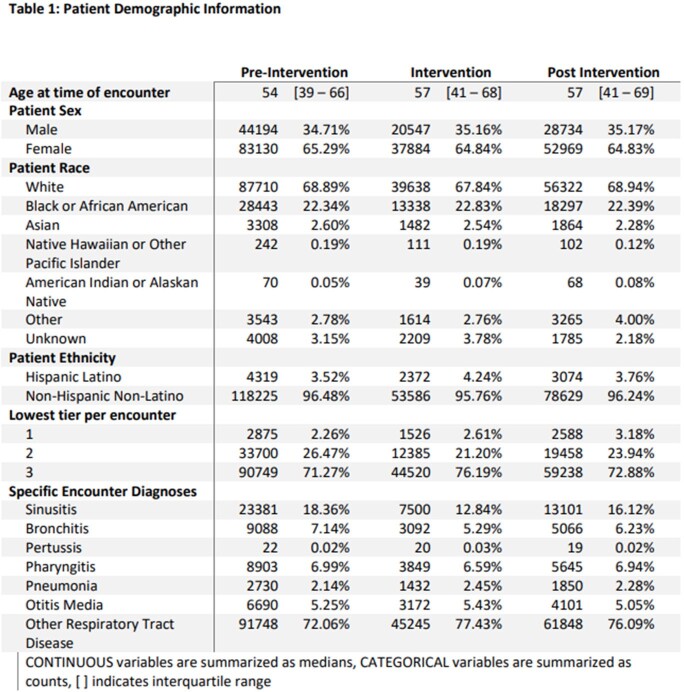

**Figure:**
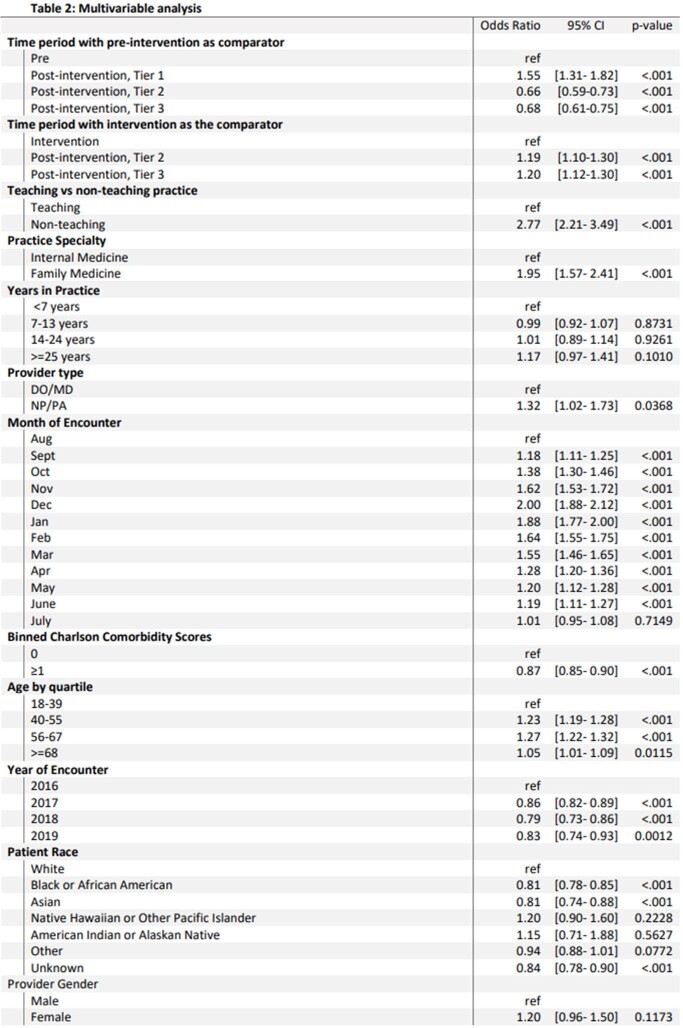

**Figure 1:**
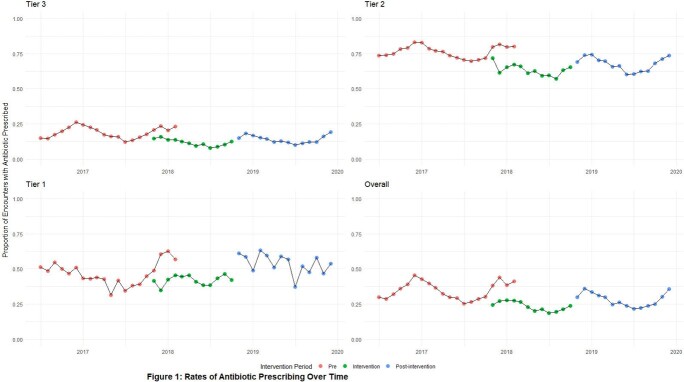
- Rates of Prescribing Over Time

